# Potential of a fly gut microbiota incorporated gel-based larval diet for rearing *Bactrocera dorsalis* (Hendel)

**DOI:** 10.1186/s12896-019-0580-0

**Published:** 2019-12-18

**Authors:** Mahfuza Khan, Kajla Seheli, Md. Abdul Bari, Nahida Sultana, Shakil Ahmed Khan, Khandokar Fahmida Sultana, Md. Anwar Hossain

**Affiliations:** 1 Insect Biotechnology Division (IBD), Institute of Food and Radiation Biology (IFRB), Atomic Energy Research Establishment (AERE), Ganak bari, Savar, Dhaka, 1349 Bangladesh; 2 Department of Microbiology, Noakhali Science and Technology University, Noakhali, 3814 Bangladesh; 3 Jashore University of Science and Technology, Jashore, Bangladesh

**Keywords:** Oriental fruit fly, Tephritidae, Gut bacteria, Gel diet, Larval rearing

## Abstract

**Background:**

The Oriental fruit fly, *Bactrocera dorsalis* (Hendel) (Diptera: Tephritidae), is an important polyphagous pest of horticultural produce. The sterile insect technique (SIT) is a proven control method against many insect pests, including fruit flies, under area-wide pest management programs. High quality mass-rearing process and the cost-effective production of sterile target species are important for SIT. Irradiation is reported to cause severe damage to the symbiotic community structure in the mid gut of fruit fly species, impairing SIT success. However, studies have found that target-specific manipulation of insect gut bacteria can positively impact the overall fitness of SIT-specific insects.

**Results:**

Twelve bacterial genera were isolated and identified from *B. dorsalis* eggs, third instars larval gut and adults gut*.* The bacterial genera were *Acinetobacter, Alcaligenes, Citrobacter, Pseudomonas, Proteus,* and *Stenotrophomonas,* belonging to the *Enterobacteriaceae* family. Larval diet enrichment with the selected bacterial isolate, *Proteus* sp. was found to improve adult emergence, percentage of male, and survival under stress. However, no significant changes were recorded in *B. dorsalis* egg hatching, pupal yield, pupal weight, duration of the larval stage, or flight ability.

**Conclusions:**

These findings support the hypothesis that gut bacterial isolates can be used in conjunction with SIT. The newly developed gel-based larval diet incorporated with *Proteus* sp. isolates can be used for large-scale mass rearing of *B. dorsalis* in the SIT program.

## Background

The insect gut contains an array of microorganisms that influence its fitness [1, 2]. Such microbial partners contribute to host metabolism [3, 4], facilitate nutrient uptake [5], prolong host lifespan [6], strengthen mating competitiveness [7], defend against natural enemies [8], and help detoxify diets [9]. Several gut bacteria have shown to act as lures [10] which may potentially be used as biocontrol agents [11, 12]. Without symbiotic bacteria, insects are reported to have reduced growth rates and higher mortality [2, 13].

Abundant symbiotic communities in the digestive tract have been reported in fruit flies including *Ceratitis capitata* (Widemann) [6, 7], *Bactrocera oleae* (Gemlin) [4, 14, 15], *Bactrocera tau* (Walker) [16, 17], *Zeugodacus* (*Bactrocera) cucurbitae* (Coq.) [18], *Bactrocera carambolae (*Drew &Hancock) [19]*, Bactrocera cacuminata* (Hering) *Bactrocera tryoni* (Froggatt) [20], the apple maggot fly, *Rhagoletis pomonella* (Walsh) [9], and the Mexican fruit fly, *Anastrepha ludens* (Loew) [21]. To characterize the gut symbiotic community structure of Tephritidae species, both culture-dependent and culture-independent approaches have been used, particularly in the med fly, which revealed a symbiotic bacterial community of different Enterobacteriaceae species from the genera *Klebsiella, Enterobacter, Providencia, Pectobacterium, Pantoea, Morganella* and *Citrobacter* [4, 22–25].

The bacterial community associated with *B. dorsalis* development is also well-studied [11, 12, 26–29]. Based on 454 pyrosequencing, the gut of different developmental stages in *B. dorsalis* harbors gut bacteria representing six phyla, where Proteobacteria dominates in the immature stages and Firmicutes (Enterococcaceae) dominates in the adult stages [30]. Using16S rRNA-based polymerase chain reaction-denaturing gradient gel electrophoresis (PCR-DGGE), the female *B. dorsalis* reproductive system revealed the presence of *Enterobacter sakazakii*, *Klebsiella oxytoca*, *Klebsiella pneumoniae*, *Raoultella terrigena* and *Enterobacter amnigenus* [11].

Explorations on other fruit fly-associated bacterial communities also revealed an almost universal presence of species-specific Enterobacteriaceae, notably species of *Enterobacter*, *Klebsiella*, and *Pectobacterium* [26, 31–33]. Strain abundance and diversity varied due to different ontogenetic stages [7, 22, 25]; however, the symbiotic community for mass rearing and genetic sexing strains (GSSs), such as the ‘Vienna 7’ strain, was reportedly reduced to only *Enterobacter* sp. [34].

The applied value of *Enterobacter* spp. in rearing *C. capitata* for the sterile insect technique (SIT) and other pest management strategies has been demonstrated in different studies [7, 13, 35, 36]. Several gut bacteria spp. (*K. pneumoniae*, *Citrobacter freundii* and *Enterobacter cloacae*) have shown to be attractive lures for Tephritidae, including *B. dorsalis* and *Bactrocera zonata* (Saunders) [10–12]. The gut bacterium, *C. freundii* of *B. dorsalis* was reported to enhance the fruit flies’ resistance to trichlorphon [37].

Encouraging results have also been reported on the use of different bacteria as probiotics (i.e., as larval or adult diet supplements) [7, 24, 36] to resolve the quality problems that may derive from disrupting the gut symbiota during mass rearing and/or irradiation [38, 39]. Supplementing *Enterobacter* sp. in the larval diet was reported to significantly enhance fitness and sexual performance of the laboratory-raised GSS *C. capitata*, ‘Vienna 8’ [40] and GSS *Z. cucurbitae* [18]. Similarly, using the med fly adult gut bacterial isolate, *K. oxytoca* as an adult diet probiotic increased the mating competitiveness of sterile mass-reared *C. capitata* males and also reduced the receptivity of wild-type females after mating with males fed the probiotic diet [7, 36].

*B. dorsalis* is a polyphagous pest species to 117 hosts, from 76 genera and 37 families in Asia [41]. The fly species causes significant economic damage to many fruits and horticultural products. SIT has been practiced as an alternative and environmentally friendly control method for *B. dorsalis* in different countries [42]. The successful use of SIT to control these fruit flies relies on mass-rearing facilities for flies with many fit, sterile adult males [39] to release irradiation-induced sterile flies in the field, targeting the *B. dorsalis* wild populations [13]. These releases lead to sterile crosses and subsequently suppress the population. However, the fruit flies targeted for SIT exhibit inferior field performance, mating competitiveness, and other qualitative parameters compared with wild fruit flies. Therefore, SIT’s success may be impaired by, artificial selection driven by mass-rearing conditions, and irradiation [7, 43].

Research conducted on *B. dorsalis* area-wide management largely focused on monitoring and control with lures [44], mating compatibility [45], spatial distribution [46], and genetics [47]. Recently, research was conducted to isolate and characterize the *B. dorsalis* gut bacterial community [11, 12, 26–29], but little is known regarding probiotic applications in *B. dorsalis* mass rearing and fitness parameters to support SIT. The present study aimed to: (1) isolate and characterize bacterial species using culture-based methods and (2) use one selected gut bacteria sp. (*Proteu*s sp.) as a dietary supplement in gel-based larval diets to assess its effects on the quality parameters of mass-reared *B. dorsalis.*

## Methods

Oriental fruit flies were obtained from a colony maintained for 60 generations on a liquid artificial larval diet [48] in the laboratory of Insect Biotechnology Division (IBD), Institute of Food and Radiation Biology (IFRB), Atomic Energy Research Establishment (AERE), Savar, Dhaka. Approximately 5000 adult flies were maintained in steel-framed cages (76.2 cm × 66 cm × 76.2 cm, H × L × W) covered with wire nets. Adults were fed protein-based diets in both liquid and dry form: (i) baking yeast: sugar: water at a 1:3:4 ratio and (ii) casein: yeast extract: sugar at a 1:1:2 ratio. Water was supplied in a conical flask socked with a cotton ball. The temperature, relative humidity and light conditions in the rearing room were maintained at 27 ± 1 °C, 65 ± 5% and a 14:10 light(L):dark(D)cycle.

### Gut bacteria isolation

Fresh eggs (6 h old, 10–15 in number), three popping (third instar) larvae, and three 15-day-old female *B. dorsalis* (reared on artificial liquid larval diet) were collected from a stock laboratory culture of the IBD. Eggs and larvae were rinsed with sterile distilled water and PBS buffer. Surface-sterilized larvae were individually dissected aseptically under a microscope. The alimentary tract was carefully removed and the mid-gut was separated with forceps and removed for analysis. Adult flies were killed by freezing at − 20 °C for 4 min. They were then surface-sterilized with 70% ethanol for 1 min, 0.5% sodium hypochloride for 1 min, washed twice in sterile distilled water and dissected to remove the gut [20].

Eggs and each gut from the *B. dorsalis* larvae and adults were placed in a sterile 1.5-ml microcentrifuge tube and washed again with sterile distilled water. All samples were homogenized separately with a sterile inoculation loop. Twenty to thirty micro liters per sample were then inoculated onto MacConkey and blood agar plates. The samples were also enriched in selenite broth. The MacConkey agar and selenite broth were aerobically incubated at 35 °C. Blood agar plates were incubated in a CO_2_ incubator at 35 °C for 24–48 h. Additional culturing was performed in BacT Alert blood culture bottles. Samples were then subcultured onto MacConkey and blood agar media and the plates were incubated as described above. All isolated colonies were sub cultured for pure growth. Bacterial isolates were initially Gram-stained to detect Gram-positive and Gram-negative bacteria along with morphology. Gram-negative rods were further identified by biochemical tests using both the conventional and Analytical Profile Index (API) 20E and 20NE (BioMerieuxsa 62,980, Marcy-1′Etoile, France) to the species level. Gram-positive cocci were identified using catalase and other related biochemical tests such as the coagulase test and later confirmed by API Strep and API Staph. ID profiles were rated from good to excellent, based on API codes (https://apiweb.biomerieux.ccom/servlet/Authenticate?action=prepare Login).

### Bacterial 16S rRNA gene amplification

Gut bacterial DNA was extracted with the ATP™ Genomic DNA Mini Kit (ATP Biotech, Inc., USA). The amount of DNA among per μl samples were measured by using Nanodrop (Thermo Scientific, USA). The 10 μl extracted DNA were amplified with 0.25 μl GoTaq® DNA polymerase (5u/μl), 10 μl 5× GoTaq® PCR flexi-buffer, 1 μl PCR nucleotide mix (10 mM each), 2 mM MgCl_2_, 1 μl (5–50 pmol) of each upstream and downstream primers and 25 μl nuclease free water in total volume of 50 μl reaction mixture. The PCR conditions were as follows: 35 cycles initial denaturation at 94 °C for 3 min, followed by 94 °C for 45 s, then annealing at 50 °C for 1 min, and an extension at 72 °C for 1 min 30 s. The amplification products (3 μl per sample) were assessed on a 1% agarose 1x Tris-acetate EDTA (TAE) gel. The detected target bands were ca. 450 bp; a negative control reaction without template DNA was used to assess the samples for contamination. The 16S rRNA gene of the representative ESBL isolates belonging to each morphological group was amplified using primers 27F and 1492R. The purified products were further used for sequencing and phylogenetic analysis. Full length sequences (1465 bp) were assembled into the SeqMan Genome Assembler (DNAstar, USA) and compared to the GenBank database of the National Center for Biotechnology Information (NCBI) (http://www.ncbi.nlm.nih.gov/GenBank) by means of the Basic Local Alignment Search Tool (BLAST) to identify close phylogenetic relatives. Five bacterial 16S rRNA partial gene sequences were isolated and deposited into GenBank (MF927674, MF927675, MF927676, MF927677 and MF927678). Multiple sequence alignment of the retrieved reference sequences from NCBI was performed using ClustalW and the evolutionary history was inferred by using the maximum likelihood method based on the Hasegawa-Kishino-Yano model [49]. Evolutionary analyses were conducted in MEGA6 [50].

### Exploitation of *Proteus* sp. as a dietary supplement in the gel-based larval diet

Once the identity of the *Proteus* sp*.* (*Proteus mirabilis*) was established by 16S rRNA gene sequencing, we selected the bacterial isolate as a probiotic dietary supplement. This isolate was derived from the gut of the *B. dorsalis* third instar larvae. Both autoclaved and live bacteria were used at the same concentrations. No bacteria were added to the control diet. To date there are no reports on using *Proteus* spp. as a probiotic on *Bactrocera* flies. *Proteus* spp. is reported to tolerate and use pollutants, promote plant growth and have potential for use in bioremediation and environmental protection [51].

### Diet formulation, preparation and delivery

The gel-based larval diet for *B. dorsalis* was prepared by adding 0.5 g agar (Sigma-Aldrich, USA) in 150 ml of liquid diet as per the modified method of Khan et al. [48]. Diet components included sugar (8.96%) (Bangladesh Sugar and Food Industry Ltd., Dhaka), soy protein (7.51%) (Nature’s Bounty, Inc., USA), sterilized wholesale soy bran (3.86%) (fine powder), baking yeast (3.77% (Fermipan red, Langa Fermentation Company Ltd., Vietnam), citric acid (1.76%) (Sigma-Aldrich, USA), sodium benzoate (0.29%), (Sigma-Aldrich, Germany), and tap water (73.85%). Initial pH for these diets was between 3.5 and 4.

Diets were prepared by weighing all ingredients and mixing them in a blender with half the water until the ingredients were fully homogenous. The agar was then mixed with the rest of the water and heated for 4 min in a microwave to boiling. After heating, the agar was added to the ingredients in the blender and mixed again until homogenous. Four-hundred-fifty ml of the gel diet were then poured into a glass beaker (500 ml) and left to cool at room temperature. Six-ml (3.8 × 10^− 6^ CFU/ml) suspensions of *Proteus* sp*.* was mixed in with the gel diet homogeneously using a magnetic stirrer and poured into the rearing tray (40 cm long × 28 cm wide × 2.54 cm deep). A small strip of wet sponge cloth (2.7 cm, Kalle USA, Inc., Flemington, NJ, USA) was placed across the middle of the gel diet, and 1.5 ml of the eggs were seeded onto the sponge using a 5-ml plastic dropper. Larval diet trays were covered with clear plastic lids until larvae began popping and began to exit the diet to pupate. The lids were then removed, and the rearing trays were placed into larger plastic containers (60 cm long × 40 cm wide × 12 cm deep) containing a 1-cm deep layer of sterile saw dust. The lid of the container had a 40-cm-diameter mesh-covered window for ventilation. Pupae were collected daily until the larvae finished jumping from the rearing tray. Three batches of experiment were conducted for autoclaved and live *Proteus* sp. treatments and the control gel-based larval diet.

### Quality parameter evaluations

The quality parameters of the flies reared on the different bacteria-added gel larval diets and the control were evaluated by assessing egg hatch (%), larval duration (days), pupal weight (mg), pupal yield (number), sex ratio (male %), adult emergence (%), flight ability (%), and survival (%) under stress. All quality parameters including survival under stress were estimated and performed under controlled laboratory condition (27 ± 1 °C, 65 ± 5% and 14 h L:10 h D).

### Egg hatch percentage

To estimate the proportion of eggs hatched, four sets of 100 eggs were spread on a 1 × 3.5 cm strip of wet blue sponge cloth and incubated in covered 55-mm Petri dishes containing the larval diets. Unhatched eggs were counted and recorded after 5 days. To calculate the mean percentage of eggs hatched, the number of unhatched eggs was subtracted from 100, then multiplied by 100.

### Larval duration

Larval duration (days) was determined by recording and collecting larvae first observed exiting from the larval diet up to 5 days of pupal collection, and estimated the mean larval period.

### Pupal weight

Pupae were collected for 5 days after larvae began exiting the diet and pupating in the saw dust. Four sets of 100 pupae per larval diet were weighed to obtain the mean weight (mg). For each larval diet, pupae from each daily collection were weighed 1 day after collection. Pupal weight (mg) from each daily collection was estimated by dividing the total weight of the pupae by the mean weight of the four sets of 100 pupae and multiplying by 100.

### Pupal yield

Pupal yield was estimated by dividing the total pupal weight (from 450 ml of each treatment diet) by the mean weight of the four sets of 100 pupae and multiplying by 100.

### Adult emergence and flight ability

Four sets of 100 pupae from the collection day with highest pupal recovery were used to assess adult emergence and the percentage of fliers. Two days before the adults emerged, four sets of 100 pupae reared on each larval diet were placed in separate 55-mm plastic Petri dish lids. The pupae dishes were then centered on 90-mm Petri dishes lined with black paper. A100-mm tall black plexi glass tube (94 mm inner diameter, 3 mm thickness) was placed on the Petri dish, and assessments were performed following previously described procedures [52]. To minimize fly-back, flies that escaped from the tube were removed daily. The flight ability test was conducted in a laboratory at 27 ± 1 °C, 65 ± 5% and a 14:10 light:dark cycle.

### Sex ratio

Four sets of 100 pupae were counted from each larval diet and placed into 1-L cylindrical plastic containers (8 cm in diameter) with a mesh section on one side (5.8 cm) for ventilation. These pupae were allowed to emerge and then scored for calculation of sex ratio.

### Effect of gut bacteria on adult survival under food and water starvation

Within 4 h of adult emergence, 25 males and 25 females were placed in a large Petri dish (70 × 15 mm) with a mesh-covered window in the lid and a hole approximately 15 mm in the center. All dishes were kept in the dark at 27 °C and 65% RH, until the last fly died. Dead flies were sorted, counted and removed from the Petri dishes on inspection twice daily (every 12 h). The surviving flies from each live and autoclaved bacteria-treated and control diet were counted.

### Statistical analysis

Within each of the three fly batches assessed, four replicates were run for each biological parameter. All data presented in this study are expressed as the mean ± standard error (SE) and were analyzed by ANOVA using Minitab, version 17. Tukey’s honest significant difference (HSD) test was used to determine significant differences among diet means.

## Results

Twelve bacterial species were isolated and identified from *B. dorsalis* eggs, third instars larval gut and adults gut*.* The common bacterial genera were *Acinetobacter, Alcaligenes, Citrobacter, Pseudomonas, Proteus,* and *Stenotrophomonas.* The physical characteristics of the *B. dorsalis* bacterial colonies at different life stages appeared similar in both culture media, with most being cream and yellow in color, while some were red. No fungi or yeasts were observed. Gram-negative and rod-shaped bacteria were the most abundant. Using API, similar gut bacterial species identified from the larval and adult guts belonged to the Enterobacteriaceae family (Table 1).

**Table 1:** Identification of *B. dorsalis* bacterial communities at different developmental stages using conventional and API methods

Developmental stages	Bacterial genera
Egg	*Acinetobacter*
	*Citrobacter*
	*Pseudomonas*
	*Stenotrophomonas*
Third instar larvae	*Acinetobacter*
	*Alcaligenes*
	*Delftia*
	*Enterobacter*
	*Pantoea*
	*Proteus*
	*Pseudomonas*
	*Staphylococcus*
	*Stenotrophomonas*
Adult	*Acinetobacter*
	*Alcaligenes*
	*Citrobacter*
	*Klebsiella*
	*Enterococcus*
	*Proteus*
	*Pseudomonas*

### 16S rRNA gene sequences

**Fig. 1:**
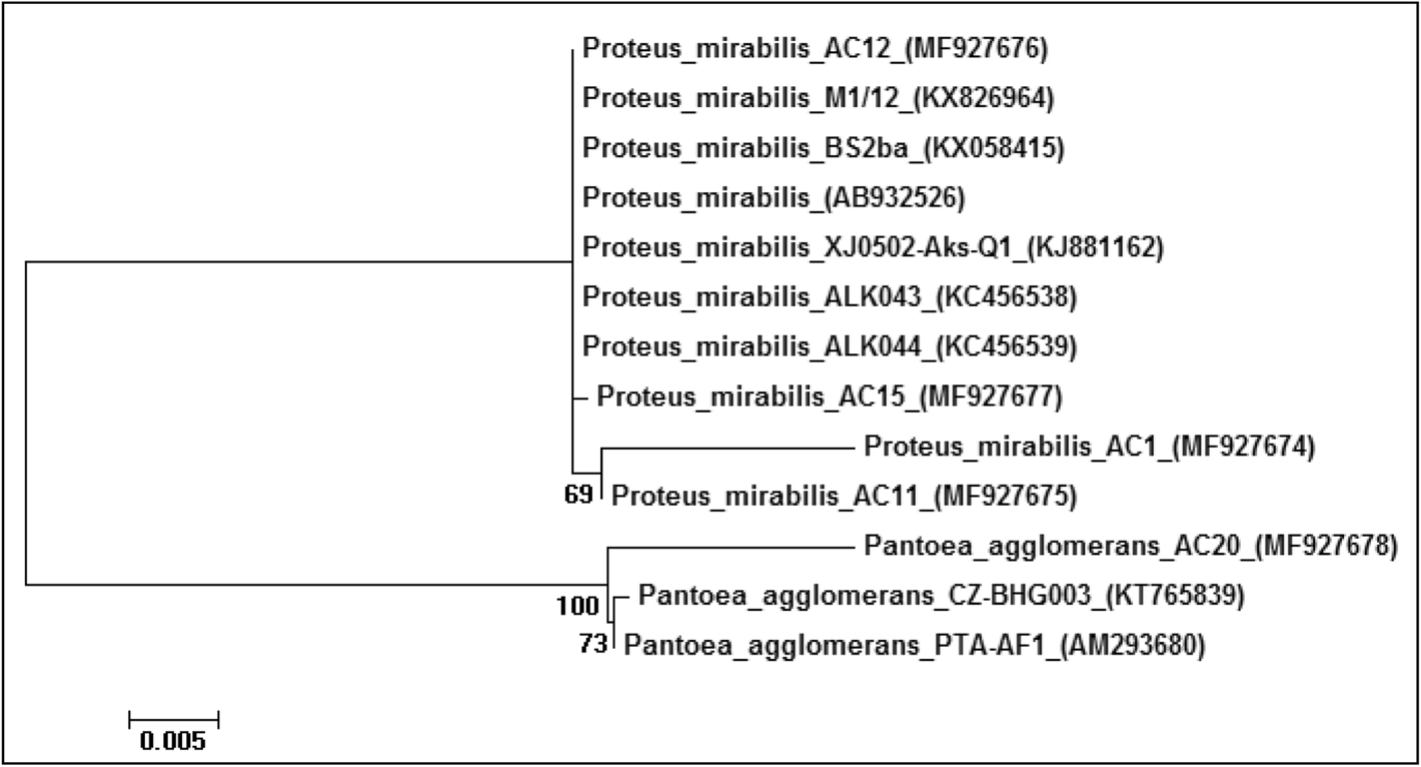
Molecular phylogenetic analysis of the *B. dorsalis* larval gut isolates by the maximum likelihood method

16S rRNA gene sequences of the bacterial isolates, AC1, AC11, AC12, AC15 and AC20, from *B. dorsalis* eggs, the guts of larvae, and adults that were isolated and identified by conventional methods and API were closely related to *Proteus mirabilis* and *Pantoea agglomerans*. Molecular phylogenetic analysis (Fig. 1) of the isolates from the *B. dorsalis* larval gut was performed by a Bootstrap consensus tree using the maximum likelihood method. The analysis involved 13 nucleotide sequences. Bootstrap values (1000 replicates) were placed at the nodes.

### Evaluation of quality parameters

The quality parameters measured for *B. dorsalis* reared on gut bacteria supplements and control gel diets are shown in Table 2.

**Table 2:** Mean (± SE) quality control parameters of *B. dorsalis* developed from live and autoclaved gut bacterial (*Proteus* sp.) supplements and control gel-based larval diets

Quality parameters	*Proteus* sp.		Control	Significance (ANOVA)
	Live	Autoclaved		
Egg hatch (%)	88.67 ± 1.33a	87.67 ± 1.66a	90.33 ± 0.88a	F = 1.02; d.f. = 2, 6; *P* = 0.415
Larval duration (days)	9.00 ± 1.15a	9.33 ± 0.88a	8.67 ± 1.45a	F = 0.08; d.f. = 2, 6; *P* = 0.925
Pupal yield (number)	9613 ± 378.58a	9379 ± 408.47a	10,129 ± 275.89a	F = 1.14; d.f. = 2, 6; *P* = 0.379
Pupal weight (mg)	10.16 ± 0.44a	10.03 ± 0.08a	10.13 ± 0.03a	F = 0.07; d.f. = 2, 6; *P* = 0.932
Adult emergence (%)	91.66 ± 0.79ab	92.33 ± 1.20a	87.33 ± 0.60b	F = 9.07; d.f. = 2, 6; *P* = 0.015
Flight ability(%)	78.33 ± 1.76a	77.33 ± 1.45a	79.66 ± 2.90a	F = 0.30; d.f. = 2, 6; *P* = 0.751
Male formation (%)	52.33 ± 2.56b	57.38 ± 0.38a	48.25 ± 4.18c	F = 28.68; d.f. = 2, 6; *P* = 0.001
Survival under stress(%)	82.00 ± 2.88a	83.00 ± 2.08a	72.33 ± 1.03b	F = 11.86; d.f. = 2, 6; *P* = 0.008

Mean (± SE) with different letters across the rows differ significantly (*P <* 0.05)

### Egg hatch percentage

Parental egg hatch was higher in the live than autoclaved *Proteus*-added diets but did not differ significantly from that of the control diet (F = 1.02; d.f. = 2, 6; *P* = 0.415) (Table 2).

### Pupal yield

Provisions of live *Proteus* sp. did not increase the *B. dorsalis* pupal yield compared to the control gel diet (F = 1.14; d.f. = 2, 6; *P* = 0.379). Autoclaved bacterial supplements did not differ significantly from the live or control diets.

### Larval duration

Diets enriched with both live and autoclaved *Proteus* sp. did not significantly reduce the duration of the *B. dorsalis* larval stage compared to the control diet. Larval stage duration for all diets ranged from 7 to 11 days and did not differ significantly among treatments (F = 0.08; d.f. = 2, 6; *P* = 0.925).

### Pupal weight

Neither live nor autoclaved *Proteus* supplements affected pupal weight (F = 0.07; d.f. = 2,6; *P* = 0.932).

#### Adult emergence and flight ability

Significantly more adults fed the live *Proteus*-treated diet emerged than those fed the control and autoclaved bacteria-treated diets (F = 9.07; d.f. = 2,6;*P* = 0.015). *Proteus* supplements did not influence flight ability (F = 0.30; d.f. = 2,6; *P* = 0.751) of *B. dorsalis* compared to those fed the control diet.

### Sex ratio

The percentage of *B. dorsalis* males was significantly higher in autoclaved *Proteus* sp. treated larval diet compared to the live *Proteus* sp. treated diet and control diet (F = 28.68; d.f. = 2,6; *P* = 0.001). However, % male from control diet was significantly lower from those of live and autoclaved *Proteus* sp. treated diets.

### Survival under stress

Longevity for the food and water deprived bacterial treatments significantly predicted adult life span (F = 11.86; d.f. = 2,6; *P* = 0.008). Survival rates of flies fed live and autoclaved *Proteus*-treated diets were higher than that of those raised on the control diet (Table 2).

## Discussion

We isolated and identified 12 bacterial genera from *B. dorsalis* eggs, third instars larval gut, and adults gut using culture-based approaches (Table 1). Using 16S rRNA techniques, we established the identity of the larval gut bacterial species, *P. mirabilis*, to test as a probiotic dietary supplement. Positive probiotic effects on *B. dorsalis* quality control parameters were recorded for percentage of adult emergence, and longevity under stress, which are important factors for SIT application. Enriching the gel-based larval diet with *Proteus* sp. improved adult emergence (92.33%), male formation (57.38%), and survival (83.00%) under stress without affecting *B. dorsalis’* egg hatching, pupal yield, pupal weight, larval duration, or flight ability compared to the control diet. Live bacteria appeared to have more potential (except percentage male) than autoclaved bacteria or the control diet (Table 2). The present gel-based larval diet appeared to be more homogenous and easier to handle when using gut bacteria as a dietary supplement for mass rearing *B. dorsalis* under controlled laboratory conditions.

*B. dorsalis* gut-associated bacterial community diversity has been reported by several authors using different isolation and characterization procedures [11, 12, 26–29]. Using next-generation sequencing of the 16S rRNA gene, a diverse group of symbiotic bacteria representing six phyla (Actinobacteria, Bacteroidetes, Cyanobacteria, Firmicutes, Proteobacteria, and Tenericutes) has been reported in the gut of *B. dorsalis* [28]. PCR-DGGE revealed the composition and diversity of the bacterial community to include *Klebsiella, Citrobacter, Enterobacter, Pectobacterium* and *Serratia* as the most representative species in adult *B. dorsalis* [26]. Based on molecular identification, *B. dorsalis* females predominantly harbored *E. cloacae*, *E. asburiae* and *C. freundii*, while *Providencia rettgerii*, *K. oxytoca*, *E. faecalis* and *Pseudomonas aeruginosa* dominated in male *B. dorsalis* [29].

In the present study, the most common genera identified in *B. dorsalis* were *Acinetobacter, Alcaligenes, Citrobacter, Pseudomonas, Proteus,* and *Stenotrophomonas.* This is consistent with previous studies that reported Enterobacteriaceae (Proteobacteria) as the most dominant family associated with tephritids [6, 7, 21–23, 25, 36, 53]; however, it contradicts recent reports that Enterococcaceae (Firmicutes) was the most dominant taxon in all life stages of *B. dorsalis* except the pupae [30]. We also recorded the presence of *Enterococcus* in the adult *B. dorsalis* gut. Andongma et al. [30] predicted that the presence of Enterococcaceae in the gut of *B. dorsalis* may help boost its immune system. However, most of the studies related to isolation and identification of gut bacterial community used adult male/female of either cultivable or wild *B. dorsalis* [12, 26, 27, 29]. Our goal was to identify cultivable bacterial species from *B. dorsalis* eggs, and larval and adult guts to identify suitable species for potential probiotic application.

The larval diet-based probiotic application of live bacteria or autoclaved *Proteus* sp. in our study did not negatively affect egg hatch, pupal yield, pupal weight, larval duration or flight ability of *B. dorsalis.* Larval diet-based probiotic application of *Enterobacter* sp., improved pupal and adult productivity and increased development by shortening the immature stages for male *C. capitata* [40]. It has been suggested that the probiotic diet’s continuous effect on med fly development might be due to *Enterobacter* sp. establishment in the larval gut supporting the host metabolism through nitrogen fixation and pectinolytic activities [4, 23].

The significantly higher emergence of *B. dorsalis* adults recorded here, using both live and autoclaved *Proteus* sp. compared to the control diet, contrasted with reports for GSS *Z. cucurbitae* [18]. *B. dorsalis* survival during limiting starvation conditions using both live and autoclaved *Proteus* sp. was significantly higher than for those reared on the control diet without probiotics. These results partly agree with those for GSS *Z. cucurbitae* where an autoclaved probiotic diet significantly enhanced adult survival rate compared with the non-probiotic diet [18]. Conversely, adult *C. capitata* survival rate on the killed probiotic diet did not differ from those reared on the ‘live probiotic’ diet [22]. Both studies noted that the autoclaved bacteria-added diet had the advantages of being more convenient and secure in handling than the live bacterial diet. In this study, the live gut bacterial species had more influence on some quality parameters of *B. dorsalis* than the autoclaved bacteria, but they did not always differ significantly from the control flies. Thus, gut microbiota usage may act on certain quality parameters of some fruit flies, while other parameters remain unaffected. However, it is difficult to compare different findings within the same species or among different fruit fly species due to the use of different bacterial strains with varying experimental conditions [7, 18, 24, 40].

The life traits of different fruit flies may be affected by diet and rearing procedures [54–57]. Several studies reported a relationship between the diet’s nutritive value and optimal development of different fruit flies such as *C. capitata, B. dorsalis, Z. cucurbitae, B. tryonii* and different *Anastrepha* species. High productivity of a gel diet in *B. tryoni* was recently reported [58] when compared with liquid [52] and solid diets. The homogeneity of different diet ingredients in the gel diet was suggested to be important in larval rearing. Here, adding the gut bacteria, *Proteus* sp. to a gel-based larval diet may have provided an additional nutrient source such as *Enterobacter* sp. [18], with more homogeneity and an increased diet ingestion rate, which eventually facilitated larvae to accumulate nutritional reserves, thus increasing adult emergence (reducing immature stage mortality), higher male production, and longevity under stress. Notably, these positive effects are important for mass rearing and large-scale SIT operational programs. Significantly more males resulted when *Proteus* sp*.* was added to the gel diet than the control diet, which might be important in supporting SIT applications since males are the active component of SIT.

Several investigations have been performed on gut bacterial manipulation during the adult stage to enhance male mating competitiveness. Irradiated ‘Vienna 8’ GSS sterile med fly males improved significantly after being fed *Klebsiella* sp. [36]; however, no increase in mating percentage of fertile male med flies after adult antibiotic treatment was observed [13]. However, mating competitiveness tests using probiotics were not performed in this study and thus require future investigation. Recent reviews [59, 60] reported the possible function of insect gut communities and their effects on fitness. To our knowledge, few studies on Tephritidae have reported adding bacteria to the larval diet [24, 40, 61] and adult food [24, 35, 36, 61, 62], and those studies were performed mainly on med flies. However, some reports conclude that gut bacteria may serve as lures and biocontrol agents in *B. dorsalis* and *B. zonata* [10–12]. However, our study showed that the gut-associated bacteria, *Proteus* sp. improved certain quality parameters in *B. dorsalis* as were reported using *Enterobacter sp.* in *C*. *capitata* [24, 40] and GSS *Z. cucurbitae* [18] larval diets. These microbiotas could be exploited to produce better quality target insects for SIT applications.

## Conclusion

The larval gut bacterial species identified during the present study through culture-based approaches belonged to the Enterobacteriaceae family. Our gel-based larval diet for mass rearing *B. dorsalis* offered opportunities for advanced laboratory studies by manipulating different nutrients and adding gut bacterial isolates. Enriching the gel diet with gut bacteria improved some *B. dorsalis* quality parameters without adversely affecting their rearing. The gut bacteria, *Proteus* sp., led to significantly more adult emergence, male formation, and survival*.* This supports the idea that probiotics can be used in conjunction with SIT. Further investigations can be performed using different macro and micronutrients (yeast products/vitamins/oils) to improve gel-based larval diets for *B. dorsalis* rearing. The effect of probiotics on mating competitiveness of *B. dorsalis* should be made in future. More beneficial gut microbiota could be exploited to produce higher quality sterile flies for SIT field application as well as for other future biotechnological applications [63].

## Data Availability

Not applicable.

## Abbreviations

AERE: Atomic energy research establishment
ANOVA: Analysis of variance
API: Analytical profile index
BLAST: Basic local alignment search tool
D: Dark
DNA: Deoxyribonucleic acid
EDTA: Ethylenediaminetetraacetic acid
ESBL: Extended spectrum beta-lactamase
GSSs: Genetic sexing strains
HSD: Honest significant difference
IBD: Insect biotechnology division
IFRB: Institute of food and radiation biology
L: Light
MEGA 6: Molecular evolutionary genetics analysis version 6.0.
NCBI: National center for biotechnology information
PBS: Phosphate buffered saline
PCR-DGGE: Polymerase chain reaction denaturing gradient gel electrophoresis
RH: Relative humidity
RNA: Ribonucleic acid
SE: Standard error
SIT: Sterile insect technique
TAE: Tris, acetate, either
